# Oral and maxillofacial surgeons’ views on the adoption of additive manufacturing: findings from a nationwide survey

**DOI:** 10.1007/s10006-024-01219-0

**Published:** 2024-02-06

**Authors:** Xuewei Zheng, Ruilin Wang, Andreas Thor, Anders Brantnell

**Affiliations:** 1 Department of Civil and Industrial Engineering, Industrial Engineering and Management, Uppsala University, Ångströmlaboratoriet, Lägerhyddsvägen 1, 752 37 Uppsala, Sweden; 2 Department of Surgical Sciences, Plastic & Oral and Maxillofacial Surgery, Uppsala University, 751 85 Uppsala, Sweden; 3 Department of Women’s and Children’s Health, Healthcare Sciences and e-Health, Uppsala University, MTC-Huset, Dag Hammarskjölds väg 14B, 1 tr, 752 37 Uppsala, Sweden

**Keywords:** Additive manufacturing, Oral and maxillofacial surgery, Surgeon’s view, Experience

## Abstract

**Objectives:**

Hospitals in many European countries have implemented Additive Manufacturing (AM) technology for multiple Oral and Maxillofacial Surgery (OMFS) applications. Although the technology is widely implemented, surgeons also play a crucial role in whether a hospital will adopt the technology for surgical procedures. The study has two objectives: (1) to investigate how hospital type (university or non-university hospital) influences surgeons' views on AM, and (2) to explore how previous experience with AM (AM experience or not) influences surgeons' views on AM.

**Materials and methods:**

An online questionnaire to capture surgeons’ views was designed, consisting of 11 Likert scale questions formulated according to the Consolidated Framework for Implementation Research (CFIR). The questionnaire was sent to OMF surgeons through the channel provided by the Association of Oral and Maxillofacial Surgery in Sweden. Data were analyzed using the Mann–Whitney U test to identify significant differences among OMF surgeons in terms of organizational form (i.e., university hospital or non-university hospital) and experience of AM (i.e., AM experience or no-experience).

**Results:**

In total, 31 OMF surgeons responded to the survey. Views of surgeons from universities and non-universities, as well as between surgeons with experience and no-experience, did not show significant differences in the 11 questions captured across five CFIR domains. However, the “individual characteristics” domain in CFIR, consisting of three questions, did show significant differences between surgeons’ experience with AM and no-experience (*P*-values: *P* = 0.01, *P* = 0.01, and *P* = 0.04).

**Conclusions:**

Surgeons, whether affiliated with university hospitals or non-university hospitals and regardless of their prior experience with AM, generally exhibit a favorable attitude towards AM. However, there were significant differences in terms of individual characteristics between those who had prior experience with AM and those who did not.

**Clinical relevance:**

This investigation facilitates the implementation of AM in OMFS by reporting on the views of OMF surgeons on AM.

## Introduction

Additive manufacturing (AM) is an alternative to mechanical manufacturing and offers several benefits in the medical field. In Oral and Maxillofacial Surgery (OMFS), AM has been in use for decades, and as demonstrated in a recent systematic review on AM in OMF surgery, many OMF surgeons today utilize digital planning and AM to enhance patient care in various treatments, including mandibular reconstruction, mandibular distraction osteogenesis, orthognathic surgery, temporomandibular joint reconstruction, facial asymmetry, and auto transplantation [1]. Multiple printing techniques are employed, each offering distinct advantages. For example, material jetting exhibits superior performance but is associated with high costs, while SLS, binder jetting, and FDM are frequently used for producing surgical training models [2]. AM provides several advantages compared to mechanical manufacturing. It enables a broader selection of materials and the production of applications with complex shapes and forms. Moreover, AM can reduce surgical time and the overall cost of care [3–6]. Still, implementation of AM in clinical practice is facing several challenges, for instance preoperative planning, image processing, implant design and manufacturing are time consuming and resource intensive [7]. In addition, new materials such as PEEK, bioprinted materials and hydrogels are needed to ensure development of AM in clinical use [8].

When introducing new technology such as AM, procedures or clinical guidelines, surgeons' views are crucial in determining whether a hospital will adopt them in patient care [9–11]. Previous studies identify both positive and negative views among surgeons. Positive views include the fact that AM provides an accurate representation when used in intraoperative planning and that it reduces surgery time [12], while negative views could stem from surgeons' perceiving AM as not a cost-effective solution [12, 13] or perceiving low advantages for their work [13]. In general, very few studies have explored surgeons' views on AM adoption, and even fewer have focused on OMFS. One exception is a recent survey from Germany reporting that surgeons are more interested in adopting AM to increase the safety of pre-surgical training by providing a “simulation-based learning environment” [13]. Previous studies have demonstrated AM as a mature technology in OMFS, with an adoption rate of around 60–65% in countries such as Germany and Sweden [13, 14].

Although showing a relatively high adoption rate (above 60%) of AM within OMFS, existing studies are inconclusive about the impact of hospital type on adoption. Evidence from Germany indicates that university hospitals were more prone to adopt AM than non-university hospitals [13], whereas a study from Sweden found no difference between these two types of hospitals in terms of AM adoption [14]. Furthermore, these studies [13, 14] did not explore how previous experience with AM influenced surgeons' views. One could hypothesize that surgeons with no experience in AM would hold more negative views on AM. To facilitate AM adoption within OMFS, it is important to comprehend how surgeons view AM and how these views are connected to hospital type and experience with AM. This study addresses this gap by examining the views of OMF surgeons on AM adoption in Sweden. In detail, the study has two objectives: (1) to investigate how hospital type (university or non-university hospital) influences surgeons' views on AM and (2) to explore how previous experience with AM (AM experience or not) influences surgeons' views on AM.

## Materials and methods

### Research design

This study is based on a quantitative survey utilizing the Consolidated Framework for Implementation Research (CFIR), which is one of the most frequently employed frameworks for studying the adoption of new treatments and guidelines in healthcare [15]. According to a recent systematic review on implementation, CFIR was used in 58% (*n* = 15) of adoption studies in healthcare [16]. CFIR comprises five main areas (domains) that influence adoption: intervention characteristics, characteristics of individuals, inner setting, outer setting, and process. The five domains in turn consists of 35 factors (constructs) that can be captured through different questions (items) [17].

### Target population and procedure

A total of 348 email addresses were provided by the Association of Oral and Maxillofacial Surgeons in Sweden, which included OMF surgeons. The target group for the survey comprised OMF surgeons undergoing clinical training and specialized OMF surgeons. Based on our experience and knowledge of the population, we estimated that this subset (OMF surgeons under training and specialized OMF surgeons) of the entire population equaled to *n* = 90. The survey was distributed via email to the respondents in April 2022, with two follow-ups during April and May 2022. The survey was administered online. The data used in this study focuses on the 11 Likert scale questions that were included in a larger survey. For further details on the population and the procedure, refer to [14].

### Data analysis

Data analysis was conducted using IBM SPSS Statistics, version 20 (Armonk, NY, IBM Corp). Likert scale questions were generated based on CFIR constructs, with responses ranging from 1 (strongly disagree) to 5 (strongly agree) in four of the five CFIR areas (individual characteristics, intervention, inner setting, outer setting). The fifth domain, process, was excluded as it primarily focuses on the planning of implementation, which is more relevant when CFIR is used to evaluate implementation processes—a goal not addressed in this study. Out of the 35 specific constructs in CFIR, we focused on 11 of them across four domains. While existing quantitative studies have often focused on just one or two areas [18], we aimed to provide a more comprehensive picture by selecting at least one construct from each of the four domains. Additionally, we focused on aspects that were frequently mentioned in previous research using CFIR (Q1-3, Q5, Q10, and Q11) [18] or were acknowledged in previous research on AM adoption (Q4, Q6-9) [3, 4] (for details, refer to Table 1).

**Table 1:** CFIR menu of constructs used to study surgeons’ views on AM in OMF surgery

CFIR		Statements
Domain	Constructs	Items
Individual characteristics	Knowledge and beliefs	Q1. AM will be an effective solution in my hospital Q2. Using AM simplifies my job Q3. AM is an important technology for the future of OMF surgery
	Individual stage of change	Q4. I am interested in adopting new technologies such as AM
	Self-efficacy	Q5. AM is or will be successfully used in my hospital
Intervention	Adaptability	Q6. The current AM technologies are suitable for my work
	Quality	Q7. Product quality is important when choosing AM
	Cost	Q8. Cost is important when choosing AM Q9. Costs are important when my patients consider choosing AM
Outer setting	Patient Needs	Q10. AM will meet the needs of the patients served by my hospital
Inner setting	Culture	Q11. New ideas are embraced and adopted in my hospital

The Mann–Whitney U test was employed to assess the differing views of surgeons from university and non-university hospitals and surgeons with and without AM experience. A significance level of p-value 0.05 was set for the comparisons. Additionally, a Cronbach reliability test was conducted to determine the correlation value between each Likert scale question for the analyzed data. A Cronbach value of 0.7 was set to assess the acceptability and reliability of the constructs.

## Result

The survey captured 31 surgeons, which covers around 34% of the target population, of these 20 (64.51%) are positioned in university hospitals, and 11 (35.49%) work in non-university hospitals. Among the surgeons, 65.5% had experience with AM and 34.5% had never used AM before. For details on the sample characteristics, refer to [14].

The survey questions were not mandatory, and some questions were not answered by all respondents. To this end, the findings were divided into two different tables for the questions where the response rates differed for the questions. Table 2 provides an overview of the response rates for the two tables (Tables 3 and 4) where response rates differed.

**Table 2:** Response rates for survey questions where response rates differed

Survey questions	University hospital	Non-university hospital
Q1-3	10	7
Q4-11	15	9

**Table 3:** University type and surgeons’ views on AM based on three items (*n* = 17)

Domain, construct and item		University hospital (*n* = 10)		Non-university hospital (*n* = 7)		*P*-value
Individual characteristics		Mean	Mean rank	Mean	Mean rank	
Knowledge and beliefs	Q1. AM will be an effective solution in my hospital	4.20	9.15	4.14	8.79	0.868
	Q2. Using AM simplifies my job	4.20	9.10	4.00	8.86	0.916
Intervention characteristics						
Adaptability	Q3. The current AM technologies are suitable for my work	3.90	8.55	4.14	9.64	0.620

**Table 4:** University type and surgeons’ views on AM based on eight items (*n* = 24)

Domain, construct and item		University hospital (*n* = 15)		Non-university hospital (*n* = 9)		*P*-value
Individual characteristics		Mean	Mean rank	Mean	Mean rank	
Knowledge and beliefs	Q4. AM is an important technology for the future of oral and maxillofacial surgery	4.27	12.13	4.33	13.11	0.722
Individual stage of change	Q5. I am interested in adopting new technologies such as AM	4.07	12.03	4.22	13.28	0.648
Self-efficacy	Q6. AM is or will be successful used in my hospital	4.07	11.80	4.33	13.67	0.497
Intervention characteristics						
Quality	Q7. Product quality is important when choosing AM	4.27	11.57	4.56	14.06	0.355
Cost	Q8. Cost is important when choosing AM	3.93	11.73	4.22	13.78	0.456
	Q9. Cost is important when my patients consider choosing AM	2.67	10.90	3.44	15.17	0.130
Outer setting						
Patient Needs	Q10. AM will meet the needs of the patients served by my hospital	3.67	12.10	3.78	13.17	0.694
Inner setting						
Culture	Q11. New ideas are embraced and adopted in my hospital	3.80	13.10	3.67	11.50	0.533

The reliability test was conducted using the Cronbach alpha test in SPSS, which tested all 11 statements indicated in Table 1. The result of the Cronbach alpha test was 0.738, which is greater than 0.7, indicating that the consistency of the data set passed the reliability test.

### Hospital type and OMF surgeons’ views on AM

Tables 3 and 4 present the results of surgeons working in two types of hospitals (university and non-university hospitals), regardless of their experience with AM. For some items, the sample sizes differ, and therefore, the data is separated into two tables. The P-values displayed in Tables 3 and 4 are all greater than the significance level, indicating that there is no significant difference between the two groups (university hospital and non-university hospital). In general, the views of the surgeons from university and non-university hospitals do not differ from each other.

### Experience of AM and OMF surgeons’ views on AM

Table 5 provides the outcomes concerning Likert scale questions Q4 to 11 with focus on previous experience of AM and OMF surgeons’ views on AM. Complete data was not obtained concerning Q1-Q3 from surgeons with no experience in AM, and thus these questions are not included in this comparison. The p-value of individual characteristics Q4-6 are smaller than the significant level, concluding that there is significant difference between the two groups (surgeons with AM experience and surgeons with no experience in AM). However, the other three domains: intervention characteristics, inner and outer settings, all have p-values greater than 0.05. concluding that there is no significant difference between the two groups.

**Table 5:** Experience of AM and surgeons’ views on AM based on eight items (*n* = 24)

Domain, construct and item		Experience of AM (*n* = 17)		No experience of AM (*n* = 7)		*P*-value
Individual characteristics		Mean	Mean rank	Mean	Mean rank	
Knowledge and beliefs	Q4. AM is an important technology for the future of oral and maxillofacial surgery	4.53	14.50	3.71	7.64	0.019*
Individual stage of change	Q5. I am interested in adopting new technologies such as AM	4.35	14.18	3.57	8.43	0.048*
Self-efficacy	Q6. AM is or will be successful used in my hospital	4.47	14.71	3.43	7.14	0.010*
Intervention characteristics						
Quality	Q7. Product quality is important when choosing AM	4.53	13.82	4.00	9.29	0.113
Cost	Q8. Cost is important when choosing AM	4.12	13.29	3.86	10.57	0.352
	Q9. Cost is important when my patients consider choosing AM	2.88	11.91	3.14	13.93	0.502
Outer setting						
Patient Needs	Q10. AM will meet the needs of the patients served by my hospital	3.82	13.38	3.43	10.36	0.294
Inner setting						
Culture	Q11. New ideas are embraced and adopted in my hospital	3.82	13.32	3.57	10.50	0.302

## Discussion

In OMFS, AM has been in use for decades across various application fields. Existing studies suggest that AM in OMFS is a mature field, with adoption rates exceeding 60% [13, 14]. However, existing studies also indicate that complete adoption still encounters several challenges, for instance, using AM in preoperative planning, image processing, implant design and manufacturing is time consuming and resource intensive [7]. When introducing new technology, like AM, surgeons' perspectives play a pivotal role in determining whether a hospital will incorporate it into patient care [9–11]. In terms of AM, one existing study, with five surgeons, found that surgeons perceive that AM provides an accurate representation when used in intraoperative planning and that it reduces surgery time [12]. The same study found that surgeons' have doubts regarding the cost-effectiveness of AM [12]. Likewise, one quantitative study found that surgeons are concerned about the cost-effectiveness and whether using AM provides advantages for their work [13]. All in all, very few studies have explicitly focused on surgeons’ when exploring adoption of AM. In this study, we examined surgeons' views on AM and explored how these perspectives were correlated with hospital types and prior experience with AM. The study yields four conclusions that hold relevance for both OMF surgeons interested in AM and hospital managers facilitating its adoption.

First, views of OMF surgeons on AM adoption do not differ between university and non-university hospitals. This is an important discovery, especially considering that previous research in a different context (Germany) had found disparities between these two types of hospitals, at least in terms of adoption rates [13]. Second, OMF surgeons in general have a positive view on AM adoption independent of hospital type. In detail, the results reveal that items related to six constructs (capturing views on AM): knowledge, beliefs, individual stage of change, self-efficacy, product quality, and cost all have mean ratings of around 4/5. Consequently, these results indicate a very positive view on AM shared by both types of hospitals. However, surgeons from both types of hospitals exhibit more hesitation towards three constructs: (1) costs, (2) patient needs, and (3) culture, with mean ratings falling below 4/5. Taking a closer look at these three constructs, we notice that the lowest-rated item, “Cost is important when my patients consider choosing AM”, is not generally in alignment with the views of surgeons from university hospitals, with a mean rating of 2.67. In contrast, non-university hospitals have a mean rating closer to 4 at 3.44. This difference cannot be attributed to the Swedish healthcare system, which covers all citizens' medical costs with government funds, resulting in patients only paying a negligible fee [19]. Therefore, it is reasonable that this item is rated relatively low. However, it is surprising that surgeons at non-university hospitals, also funded by public means, rate this item higher. Nevertheless, the difference is not statistically significant. The second item, rated below 4 by both types of hospitals, “AM will meet the needs of the patients served by my hospital”, suggests that despite the assumption that AM should provide a better fit for patient needs [20], there is still room for improvement in terms of alignment with patient needs. The third item, rated below 4 by both types of hospitals, “New ideas are embraced and adopted in my hospital”, indicates that there is room for improvement in terms of hospital support for the adoption of new technology such as AM. Overall, the high ratings for all eight constructs and eleven items (with only one item rated below 3) are somewhat surprising, given that German OMF surgeons in previous research were hesitant about the usefulness of AM for their work [13]. These findings are also supported outside AM adoption, in other clinical fields concerning adoption of new technology, where the usefulness of the new technology was a concern for adoption [21]. These somewhat negative views on AM and new technology are not evident among Swedish OMF surgeons. Future research could investigate whether there are differences in views of OMF surgeons on AM adoption in countries outside Sweden.

Third, views of OMF surgeons on AM adoption are similar between those with experience of AM and those who lack experience in terms of constructs related to quality, cost, patient needs and culture. This is an interesting finding since one could assume that surgeons with no experience in AM would hold more negative views on AM since we know from existing research that those who have not tried a new technology after several years are most likely late adopters or laggards that often are skeptical about the technology [22]. Our findings indicate that with OMF surgeons and AM this is not the case. Future research could investigate if late adopters or laggards within OMF surgery in countries outside Sweden hold positive views on AM although not having experience of it. Fourth, there are significant differences between those with experience of AM and those who lack experience in terms of individual characteristics. In detail, three constructs under individual characteristics (knowledge and beliefs, individual stage of change, and self-efficacy) all have significant p-values (*p* < 0.05). The surgeons who have experience with AM believe AM is worth investing in, have more interest in using other new technologies in the future, and believe in their capability of handling the technology if it is successfully implemented in their hospitals. In contrast, surgeons that lack experience of AM are more conservative and hesitant concerning AM. These findings imply that increasing possibilities for surgeons to experiment with AM could influence their views on AM in terms of knowledge and beliefs, individual stage of change and self-efficacy, and thus might in turn increase their use of AM.

## Conclusions

OMF surgeons, in both university and non-university hospitals in Sweden and those with experience and those lacking experience, show a very positive view of AM technology, indicating a relatively high acceptance of new technologies such as AM. However, surgeons who have experience of AM have more positive views on AM in terms of individual characteristics such as interest to use AM and promises of the technology when compared to surgeons who lack experience of AM. Some limitations should be raised. First, the survey was administered on a voluntary basis, and participants were not obligated to answer all questions. As a result, there were variations in the data size for different items. Although having complete data would have been ideal for a thorough analysis, this approach was taken due to a strategic consideration. Surgeons in this field often have demanding schedules, making it challenging to secure their participation. Second, it is worth noting that there is a likelihood that the respondents who participated in this research might have been inclined to answer and thus do not represent the entire population of OMF surgeons in Sweden. Conversely, surgeons who have never used or are less familiar with AM may have been less inclined to respond. Nevertheless, it's important to highlight that this study encompasses data from both those who have experience and those who do not. As a result, it is expected to offer a comprehensive perspective on the adoption of AM within OMFS in Sweden.

## Data Availability

The data supporting the findings of this study are available within the article.
